# Spatial Spillover Effects of Digital Infrastructure on Food System Resilience: An Analysis Incorporating Threshold Effects and Spatial Decay Boundaries

**DOI:** 10.3390/foods14091484

**Published:** 2025-04-24

**Authors:** Yani Dong, Chunjie Qi, Cheng Gui, Yueyuan Yang

**Affiliations:** 1 College of Economics and Management, Huazhong Agriculture University, Wuhan 430070, China; dongyani@webmail.hzau.edu.cn (Y.D.); guicheng@webmail.hzau.edu.cn (C.G.); yangyueyuan@webmail.hzau.edu.cn (Y.Y.); 2 Institute of Horticultural Economics, Huazhong Agriculture University, Wuhan 430070, China; 3 Hubei Rural Development Research Center, Wuhan 430070, China

**Keywords:** application of digital technologies, food security, direct effect, indirect effect, total effect, degree of market integration, attenuation boundary analysis

## Abstract

As an important carrier for the application of digital technologies, digital infrastructure plays a crucial role in promoting the digital transformation of the grain system and ensuring food security in the current era. This study utilizes panel data from 31 provinces (municipalities) in China, spanning the years from 2006 to 2022, and constructs a comprehensive evaluation index system for grain system resilience, grounded in its core components of resistance, recovery, and transformation. The grain system resilience index is measured using the entropy method. A spatial Durbin model is employed to estimate the impact of digital infrastructure on grain system resilience, and a panel threshold model is used to analyze the nonlinear effects of digital infrastructure on grain system resilience. The research findings are as follows: (1) Both the direct and spatial spillover effects of digital infrastructure on grain system resilience are significantly positive, but considerable regional heterogeneity is observed. Due to differences in economic development levels, digital infrastructure investments, and policy priorities, the indirect and total effects of digital infrastructure on food system resilience are more pronounced in the southeast region, whereas the direct effects are more significant in the northwest region. (2) The threshold regression results show that when market integration is below the threshold value, the estimated coefficient of digital infrastructure is 0.2242, which is significant at the 1% significance level. When market integration is above the threshold value, the estimated coefficient of digital infrastructure is 0.0790, which is also significant at the 1% significance level. However, its regression coefficient significantly decreases, indicating that the impact of digital infrastructure on food system resilience will weaken as the degree of market integration increases. (3) The analysis of the attenuation boundary of spatial spillover effects shows that within a distance of 225 km, the estimated coefficients of the indirect effects of digital infrastructure on grain system resilience are positive and statistically significant at least at the 10% significance level. However, beyond 225 km, the regression coefficients become negative and insignificant, indicating that the effective boundary of the spillover effect of digital infrastructure on grain system resilience is 225 km, after which the spillover effect gradually diminishes. Based on these findings, it is recommended that the southeast region further strengthen regional digital governance collaboration to maximize spillover effects, whereas the northwest region should prioritize improving digital infrastructure and introduce digital technologies through models such as an “enclave economy” to bridge the digital divide. This study reveals the impact of digital infrastructure on grain system resilience and provides a new perspective for scientifically evaluating the spatial spillover effects of digital infrastructure.

## 1. Introduction

The report of the 20th National Congress of the Communist Party of China emphasizes “ensuring the security of food, energy resources, and important industrial and supply chains.” However, multiple internal and external challenges, such as tightening resource and environmental constraints, the spiraling upward trend of short-term costs of production factors, increasingly frequent extreme weather events, the rise of trade protectionism, and persistent geopolitical conflicts, are compounding and striking the global food industry and supply chains. This has led to increased uncertainties and instabilities in the food system, with prominent issues of insufficient resilience. Studies integrating population dynamics, food production, and trade indicate that the global food system is becoming increasingly fragile [1,2,3,4,5]. According to the 2024 Global Food Crisis Report, intensifying conflicts, extreme weather, and economic shocks have resulted in approximately 282 million people in 59 countries and regions facing severe food insecurity in 2023, an increase of approximately 24 million people compared to 2022. The situation of severe food insecurity globally continues to deteriorate [6]. Undoubtedly, this has drawn widespread attention from governments and scholars around the world, including China, to the resilience of agricultural and food systems [7,8,9]. For example, the Food and Agriculture Organization of the United Nations (FAO) pointed out in the 2021 State of Food and Agriculture report that “building resilient agricultural and food systems is a strategic element for the world to address current and future challenges” [10]. At the 26th United Nations Climate Change Conference, Qu D.Y., Director-General of FAO, called for “accelerating the transformation of agricultural and food systems towards greener, more inclusive, resilient, efficient, and sustainable directions to achieve the global goal of zero hunger by 2030” [11]. In addition, some scholars believe that enhancing the resilience of food systems is not only an inevitable choice for ensuring food security but also a practical measure to achieve multiple Sustainable Development Goals [12,13]. Against this backdrop, there is an urgent need to accelerate efforts aimed at steadily improving food system resilience.

In recent years, scholars have conducted research on the connotation, measurement methods, and influencing factors of food system resilience. Regarding the connotation of food system resilience, Seekell et al. (2017) argue that the ability of a food system to respond to and adapt to shocks while maintaining its functions is an inherent manifestation of food system resilience [14,15]. Zurek et al. (2022) define food system resilience as the ability of the food industry to maintain stability, self-adjust, and repair itself when subjected to various natural or non-natural internal and external shocks. It is a comprehensive capability that encompasses individuals, local governments, nations, and the global level [16,17]. Meuwissen et al. (2020) further proposed assessing the resilience of agricultural and food systems by distinguishing three types of resilience capacities: resistance capacity, which refers to the ability to withstand stress and shocks; recovery capacity, which refers to the ability to change in response to shocks without altering the system’s structure and feedback mechanisms; and transformation capacity, which refers to the ability to change the system’s structure and feedback mechanisms in the face of shocks or stress [18]. In terms of measurement, like all complex social–ecological systems, food system resilience cannot be evaluated on a single scale [19]. It must include interactions at local, global, and cross-scales [20,21,22]. The Food and Agriculture Organization of the United Nations (FAO) proposed national-level indicators for agricultural food system resilience in its 2021 report, The State of Food and Agriculture 2021, including the Primary Production Flexibility Index, the Dietary Source Flexibility Index, the Midstream Flexibility Index, and the Resilience Capacity Index [10]. Some studies have also measured agricultural resilience by comparing the actual growth path of agricultural labor productivity with its predicted counterfactual growth path [23]. Regarding influencing factors, scholars believe that factors such as crop diversification [24,25], infrastructure construction [26,27], and the application of digital technologies [28] play a key role in enhancing food system resilience. Additionally, the growth of total factor productivity (TFP) in agriculture is also considered an effective way to improve food system resilience. For example, Coomes et al. (2019) pointed out that the growth of agricultural TFP can promote sustainable agricultural development, thereby enhancing agricultural resilience [29].

In fact, the gradual improvement in digital infrastructure and the rapid development of digital technologies such as 5G, big data, and industrial internet has provided significant benefits to the development of the food industry. From the perspective of food production, Nakasone et al. (2014) found that information and communication technology (ICT) not only enhances farmers’ understanding of agricultural practices and technologies but also improves the efficiency of agricultural production [30]. Weltzien (2016) argued that interconnected digital tools can provide new momentum for agricultural development, benefiting the environment, biodiversity, and farmers [31]. Wolfert (2017) suggested that digital technology can provide farmers with optimal production solutions, thereby improving production efficiency [32]. Alam et al. (2023) also suggested that the use of ICT can overcome many challenges in the agricultural food system, including maintaining precise farm management, product marketing, and access to production inputs, which is conducive to enhancing the resilience of the agricultural food system network [33]. From the perspective of food supply, some studies argue that the essence of digital technology’s impact on food supply chain resilience lies in its application across various stages of the food supply chain, including production, storage, processing, transportation, and sales. This enhances resource allocation efficiency, identifies weak points with precision, and improves supply chain resilience [34,35].

Although existing studies have widely recognized the promoting role of digital technology in food production or supply, there has been no empirical analysis examining the relationship between digital infrastructure and food system resilience. Moreover, the impact of digital infrastructure on food system resilience from the perspective of spatial spillover effects has been overlooked.

In light of this, this study uses panel data from 31 provinces (municipalities) in China from 2006 to 2022 to construct an indicator system for food system resilience based on three dimensions: resistance capacity, recovery capacity, and transformation capacity. A spatial econometric model is employed to analyze the impact and mechanisms of digital infrastructure on food system resilience. The potential innovations of this study are as follows: First, considering the spatial externality of digital infrastructure, a spatial econometric model is constructed to study its impact on food system resilience. Second, although previous studies have emphasized the promoting role of digital infrastructure in food production or supply, this study argues that the impact of digital infrastructure on food system resilience is constrained by market integration. Therefore, a panel threshold model is used to further analyze the impact of digital infrastructure on food system resilience. Third, considering the factor of geographic distance, this study examines whether the impact of digital infrastructure on food system resilience changes with geographic distance.

## 2. Theoretical Analysis and Research Hypothesis

### 2.1. Digital Infrastructure, Direct Effects, and Food System Resilience

Digital infrastructure has played a positive role in promoting improvements in food system resilience. Firstly, the digital transformation of the grain industry driven by digital technology enables precise matching between the supply and demand sides of grain production factors, facilitating the deep integration of digital technologies and agriculture. This not only enhances the efficiency of grain production [36] but also reshapes the production, management, and industrial systems of the grain industry [37]. Secondly, the development of digital infrastructure is conducive to enhancing the extensibility of financial services [38]. This infrastructure can overcome the spatiotemporal limitations of traditional financial services and reduce the “financial exclusion” of agricultural business entities. This effectively alleviates the information and financing constraints faced by entities in the grain industry [39]. Moreover, it can broaden the capital element channels for grain industry development, assisting business entities in using financial tools for risk management and control [40]. Finally, interconnected digital tools can provide new momentum for grain industry development. Integrating data resources in each link of the grain industry chain and expanding the application scenarios of digital technologies throughout the entire grain industry chain, including production, storage, processing, transportation, and sales [34], promotes the transformation of grain production toward “intelligence”, “refinement”, and “greening”, reducing the sensitivity and exposure of the grain production end to natural risks. These systems can also regulate the relationship between grain production and consumption, achieving interconnectivity in the production, circulation, trading, and service links of the grain industry [41]. This extends and expands the grain industry chain, thereby enhancing food system resilience.

**Hypothesis 1:**  Digital infrastructure has a direct promoting effect on food system resilience.

### 2.2. Digital Infrastructure, Spatial Spillover Effects, and Food System Resilience

Digital infrastructure not only affects the food system resilience of the local area but also impacts that of neighboring regions. Compared with traditional infrastructure, digital infrastructure can overcome time and space limitations; accelerate the dissemination and exchange of technology, knowledge, and information among regions; and promote the free flow of factor resources between regions. It thus has strong spillover and external characteristics [42]. Therefore, digital infrastructure may also have a significant spatial spillover effect on the food system resilience of neighboring regions. On the one hand, the “halo effect” generated by local digital infrastructure can have a positive impact on the food system resilience of neighboring regions. Given the relatively short spatial distance, neighboring regions are more likely to learn from the advanced experiences and models of the local area. The improvement of food system resilience in the local area can serve as a leading and demonstrative role for neighboring regions. On the other hand, local digital infrastructure may also lead to an expansion of the digital divide, yielding a negative impact on the food system resilience of neighboring regions. Specifically, when the development of digital infrastructure in neighboring regions lags behind that of the local area, it may exacerbate the problem of information asymmetry between regions. Additionally, it may weaken the development potential of neighboring regions due to the siphon effect [43]. Meanwhile, if neighboring regions overly rely on the digital infrastructure and services of the local area—such as major grain-producing provinces like Heilongjiang and Liaoning, which are limited by their economic development levels and find it difficult to effectively bear the construction and maintenance costs of big data carrier platforms for the grain industry chain [44]—they may depend on digital platforms or services in other regions. This could increase the risk conduction effect between regions, thus exerting negative external pressure on neighboring regions. In summary, digital infrastructure has positive or negative spatial spillover effects on the food system resilience of neighboring regions. Based on this, this study proposes Hypothesis 2.

**Hypothesis 2:**  The spatial spillover effect of digital infrastructure on food system resilience is uncertain and may be either a positive or a negative spillover effect.

### 2.3. Digital Infrastructure, Threshold Effects, and Food System Resilience

The promotion of food system resilience by digital infrastructure depends to a certain extent on the degree of marketization. The improvement of market integration can eliminate geographical and administrative boundaries, break local protectionism, reduce market entry thresholds, and lower transaction costs. Through competitive and matching effects, this improvement can enhance the efficiency and quality of the allocation of various production factors, such as labor, technology, and capital. This strengthens cooperation and communication among different links of the industrial chain and promotes the coordinated development of regional industrial chains [45], which is conducive to enhancing the overall competitiveness of the grain industry chain. Theoretically, digital infrastructure is conducive to promoting the development of the market into a unified large market with perfect competition [46]. However, excessive market integration may strengthen market concentration and trigger new monopoly risks [47], thus posing potential threats to the grain industry chain. Based on this, this study proposes Hypothesis 3.

**Hypothesis 3:**  Market integration has a significant threshold effect on the extent to which digital infrastructure promotes food system resilience.

**Figure 1:**
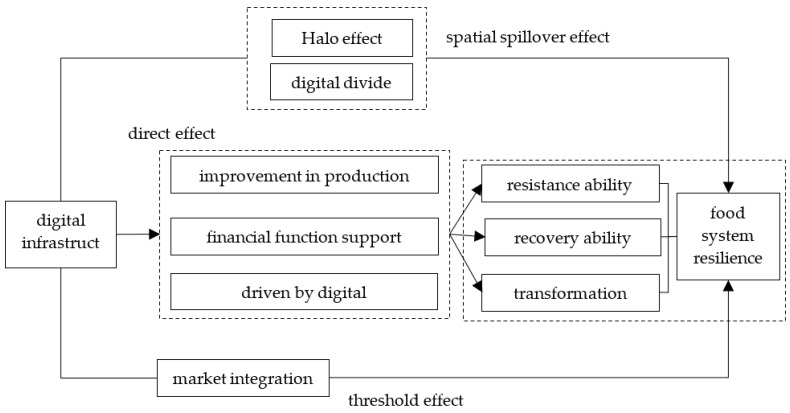
Theoretical analysis framework of the impact of digital infrastructure on food system resilience.

Based on the above analysis, this study constructs a theoretical analysis framework to assess the impact of digital infrastructure on food system resilience (Figure 1).

## 3. Methods and Data Description

### 3.1. Methods

#### 3.1.1. Entropy Value Method

This study employed the entropy value method to measure food system resilience. Firstly, to eliminate the influence of differing measurement units during the calculation of the entropy value method, different standardization methods are applied to positive and negative indicators in the original dataset. Secondly, the proportion of each sample value relative to the total value of the corresponding indicator was calculated. Based on these proportions, the entropy value and variation coefficient of each indicator were computed to determine the indicator weights. Finally, a comprehensive score was obtained based on the weights and the standardized data. The specific calculation steps are as follows:

Step 1: Standardize the data.

The formula for positive indicators is expressed as follows:

(1) $$X^{\prime }=\frac{X_{ij}-min(X_{ij})}{max(X_{ij})-min(X_{ij})}$$

The formula for negative indicators is expressed as follows:

(2) $$X\prime =\frac{max(X_{ij})-X_{ij}}{max(X_{ij})-min(X_{ij})}$$

Step 2: Calculate the proportion of the i province’s j indicator to the total of that indicator.

(3) $$P_{ij}=\frac{X_{ij}}{\sum_{i=1}^{M}X_{ij}}$$

Step 3: Calculate the entropy values of the j indicators.

(4) $$e_{j}=\frac{1}{ln(M)}\cdot \sum_{i=1}^{M}\left(P_{ij}\cdot lnP_{ij}\right)$$

Step 4: Calculate the difference coefficient of the j indicator.

(5) $$d_{j}=1-e_{j}$$

Step 5: Calculate the weights of the evaluation indicators.

(6) $$W_{j}=\frac{d_{j}}{\sum_{j=1}^{N}d_{j}}$$

Step 6: Calculate the comprehensive score of the evaluation indicators.

(7) $$Z_{i}=\sum_{j=1}^{N}W_{j}•X_{ij}$$

#### 3.1.2. Spatial Econometric Model

This study adopted a spatial econometric model to explore the impact and spatial effects of a digital infrastructure on food system resilience. The application of spatial econometric models mainly includes the spatial lag, spatial error, and spatial Durbin models. Because the spatial Durbin model takes into account both the spatial dependence of the explanatory variables and the spatial lag term of the dependent variable, it can simultaneously reflect spatial autocorrelation effects and spatial spillover effects. Therefore, the following spatial Durbin model was constructed:

(8) $$\begin{array}{cc} resi_{it}= & \beta_{0}+\rho W_{ij}resi_{it}+\beta_{1}W_{ij}dig_{it}+\beta_{2}dig_{it}+\\ & \beta_{3}W_{ij}controls_{it}+\beta_{4}controls_{it}+\gamma_{i}+\delta_{t}+\varepsilon_{it} \end{array}$$

Here, $resi_{it}$ represents the food system resilience of province i in year t; $dig_{it}$ represents the digital infrastructure of province i in year t; $controls_{it}$ represents the control variables; $W_{ij}$ is the spatial weight matrix; ρ is the spatial autoregressive coefficient to be estimated; $\beta_{0}$ is the constant term; $\beta_{1},\beta_{2},\beta_{3},\beta_{4}$ are the parameters to be estimated; and $\gamma_{i}$, $\delta_{t}$, and $\varepsilon_{it}$ represent individual effects, time effects, and random disturbance terms, respectively.

Because the spatial inverse distance matrix is a weight matrix constructed based on spatial location relationships, it can represent the mutual influence between any two provinces. The closer the distance is, the more obvious the effect. Therefore, this study used the spatial inverse distance matrix for regression analysis, which is expressed as follows:

(9) $$W_{ij}=\left\{\begin{array}{l} 1/d_{ij},i\neq j;\\ 0,i=j \end{array}\right.$$

### 3.2. Variable Selection

#### 3.2.1. Dependent Variable

This study took the resilience of the grain system as the explanatory variable; it is denoted as Resi. Based on China’s national and agricultural conditions, in this paper, grain system resilience refers to the resistance, recovery, and transformation abilities demonstrated by various links, such as grain production, storage, transportation, processing, and consumption when facing uncertainty shocks. It not only includes the resilience of the grain production link but also moderately takes into account the resilience of the entire process, including circulation, processing, and consumption. Refer to the existing research [10,48,49], and centering around the three dimensions of resistance, recovery, and transformation displayed by the grain industry chain in response to external shocks, an evaluation system for grain system resilience was designed (see Table 1). Specifically, the resistance ability covers three secondary indicators: basic guarantee, supply stability, and risk control. Grain production depends on arable land, labor, and irrigation infrastructure, and is crucial for the entire grain system. Meanwhile, grain supply stability includes stable grain transportation and market stability because an efficient transportation and distribution network and a stable grain market can enhance the allocation ability of the grain system in the face of risks. In addition, grain production is also vulnerable to disasters, so risk control is necessary. Given data availability limitations, agricultural insurance-related data are used to measure risk control. The recovery ability includes two key elements: recoverability and sustainability. In terms of recoverability, the multiple cropping index reflects the efficiency of land use and production continuity as well as the land’s ability to quickly restart production after interference. The growth rate of the total agricultural output value comprehensively reflects the recovery and growth potential of the agricultural economy after being hit, which involves the coordination of multiple elements; the income level of farmers is related to their production input and enthusiasm and is also an embodiment of rural economic vitality; and the rural medical level ensures the health of the labor force and stabilizes the social structure, creating conditions for the recovery of grain production. In terms of sustainability, the intensity of fertilizer and pesticide application in grain production reflects the efficiency of resource utilization and the impact on the ecological environment. Moderate use is conducive to long-term stability; the disaster incidence rate of grain crops measures the stability and risk resistance of the system. A decrease in the disaster incidence rate indicates enhanced response and recovery capabilities. These indicators jointly ensure the sustainable development of the grain system. The transformation ability includes four elements: industrial synergy, innovation synergy, government synergy, and financial synergy. The rational allocation of grain production factors and the in-depth integration of the primary, secondary, and tertiary industries of grain play important roles in promoting the optimization and upgrading of the grain industry structure. Therefore, the grain processing and service industries must be taken into account. Technological innovation is essential for enhancing the quality and efficiency of the grain system, as it reflects the grain system’s capacity for technological advancement and the application of innovative practices. In addition, fiscal expenditure and financial mechanisms can provide crucial support for the grain system, facilitating its transformation.

Furthermore, using Formulas (1)–(6), the weights of the primary indicators, secondary indicators, and each individual indicator related to the resilience of the grain system were calculated. The weight results are reported in Table 1. Specifically, regarding primary indicators, the resistance ability has the highest weight, accounting for 0.6449, followed by the transformation ability, with a weight of 0.3132, and finally the recovery ability, with a weight of 0.0420. Regarding secondary indicators, the top four in terms of weight are supply stability, risk control, industrial synergy, and basic guarantee, with weights of 0.2773, 0.2010, 0.1942, and 0.1666, respectively. The weights of the remaining secondary indicators do not exceed 0.1. Generally speaking, the high weight of resistance ability reflects the core contradiction of food security. The resistance ability of the grain system is crucial when facing shocks. When the shocks exceed the carrying capacity of the grain system, its ability to recover and transform loses its foundation for effective action. The second-highest weight of the transformation ability also reflects the long-term value of grain system transformation. The relatively low weight of the recovery ability may be due to the strong seasonality of the grain system. After a shock occurs, there may be time constraints, and environmental protection measures also take time to materialize. This suggests that to enhance the resilience of the grain system, priority should be given to strengthening its resistance and improving its transformation capabilities to ensure grain production and supply stability. At the same time, it is necessary to reduce environmental damage in the long term to ensure sustainable development.

#### 3.2.2. Core Explanatory Variable

The core explanatory variable in this study is digital infrastructure, which is denoted as Dig. A rich body of literature on the measurement of digital infrastructure is available. Most scholars have used the “Broadband China” strategy or the “Smart China” strategy as proxy variables for digital infrastructure. However, due to potential differences in policy implementation during the execution of these strategies, this study referred to research by Li Yunhe et al. (2022) [50] and adopted the per capita number of Internet broadband access ports, a more objective indicator, to measure digital infrastructure. The data were sourced from the WIND database.

#### 3.2.3. Control Variables

To further control for internal and external factors that may interfere with the impact of digital infrastructure on food system resilience, this study selected the following control variables:

Rural Population Aging, Denoted as Aging: The continuous deepening of rural population aging is often accompanied by a decline in both the quantity and the quality of the agricultural labor supply, leading to a gradual shift in agricultural production toward an “elderly-dominated agriculture” model [51]. This variable was included to account for the impact of changes in grain production inputs on food system resilience. Referring to the study by Yu Y.Q. et al. (2024) [52], it was measured as the proportion of the rural population aged 65 years and above to the total rural population.

Agricultural Development Level, Denoted as Adev: Theoretically, the higher the level of regional agricultural development is, the more complete and efficient the grain industry chain is, which is more conducive to the grain industry chain’s ability to respond to risk shocks. Referring to the study by Wang S.H. et al. (2024) [53], it is represented by the proportion of the total output value of the primary industry to the regional gross domestic product (GDP).

Grain Fixed Asset Investment, Denoted as Inve: Increasing rural fixed asset investment has a positive effect on improving grain yield per unit area and increasing cultivated land area. It helps to improve agricultural labor efficiency and enhances farmers’ ability to resist natural disasters [54]. It is estimated by multiplying the ratio of grain output value to the total output value of agriculture, forestry, animal husbandry, and fishery by the fixed asset investment in agriculture, forestry, animal husbandry, and fishery.

Rural Power Facilities, Denoted as Elec: Rural power facilities are essential conditions and primary guarantees for the production and life of rural residents, making a significant contribution to improving total factor productivity in agriculture [55]. Referring to the study by Hao Aimin et al. (2023) [56], it was measured as the proportion of rural electricity consumption to the total rural population.

Educational Attainment of Rural Labor Force, Denoted as Educ: An increase in the educational attainment of rural residents is conducive to enhancing their ability and efficiency in acquiring, understanding, and accepting production information, thereby promoting the improvement of technical efficiency in agricultural output [57]. Referring to the study by Dong Y et al. (2018) [58], the per capita educational attainment was used as a proxy variable for the educational attainment of the rural labor force. The formula for per capita educational attainment is expressed as follows: (years of no schooling × 0 + years of primary school × 6 + years of junior high school × 9 + years of senior high school × 12 + years of college and above × 16)/total population aged 6 and above.

Table 2 reports the descriptive statistics of all variables.

### 3.3. Data Sources

This study used panel data from 31 provinces, autonomous regions, and municipalities directly under the Central Government of China (hereinafter referred to as “provinces”, excluding the Hong Kong, Macao, and Taiwan regions) from 2006 to 2022 as a sample for analysis. The data were sourced from the National Bureau of Statistics of China, the EPS Database, the Wind Database, the Zhejiang University—China Agriculture-Related Research (CARD) Database, the Zhihuiya Patent Database, and the annual statistical yearbooks of China, including the China Statistical Yearbook, China Rural Statistical Yearbook, China Tertiary Industry Statistical Yearbook, China Finance Yearbook, China Population and Employment Statistical Yearbook, China Rural Cooperative Management Statistical Yearbook, and China Rural Cooperative Economy Statistical Yearbook, as well as the statistical yearbooks of each province.

## 4. Analysis of Research Results

### 4.1. Spatial Autocorrelation Test

#### 4.1.1. Global Autocorrelation

The prerequisite for using spatial econometrics is the existence of spatial dependence between digital infrastructure and food system resilience. Therefore, the Moran’s index was used to test spatial autocorrelation. Its calculation formula is provided below:

(10) $$Moran\prime I=\frac{\sum_{i=1}^{n}\sum_{j=1}^{n}W_{ij}\left(x_{i}-\bar{x}\right)\left(x_{j}-\bar{x}\right)}{S^{2}\sum_{i=1}^{n}\sum_{j=1}^{n}W_{ij}}$$

(11) $$S^{2}=\frac{1}{n}{\sum_{i=1}^{n}\left(x_{i}-\bar{x}\right)}^{2},\bar{x}=\frac{1}{n}\sum_{i=1}^{n}x_{i}$$

Here, $S^{2}$ is the sample variance; $x_{i}$ and $x_{j}$ represent the observations of the i and j regions, respectively; n is the number of sample regions; and $W_{ij}$ is the spatial weight value between regions.

Table 3 reports the global Moran’s indices of digital infrastructure and food system resilience. According to Table 3, from 2006 to 2022, the Moran’s indices of digital infrastructure and food system resilience were both greater than 0, and the *p*-values were both less than 0.05. These findings indicate a positive spatial autocorrelation between digital infrastructure and food system resilience. Therefore, a spatial econometric model can be used to analyze the impact of digital infrastructure on food system resilience.

#### 4.1.2. Local Autocorrelation

To investigate the local spatial agglomeration status of digital infrastructure and food system resilience, local Moran scatter plots were generated.

**Figure 2:**
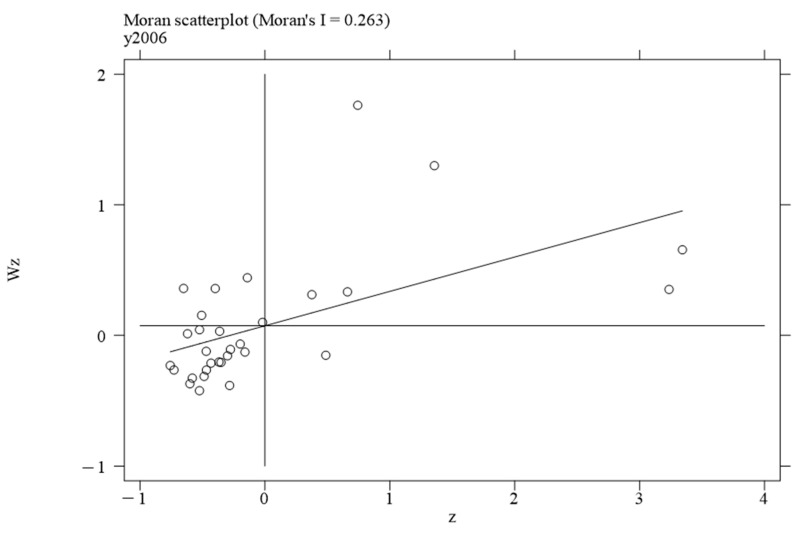
Moran scatterplot of digital infrastructure in 2006.

**Figure 3:**
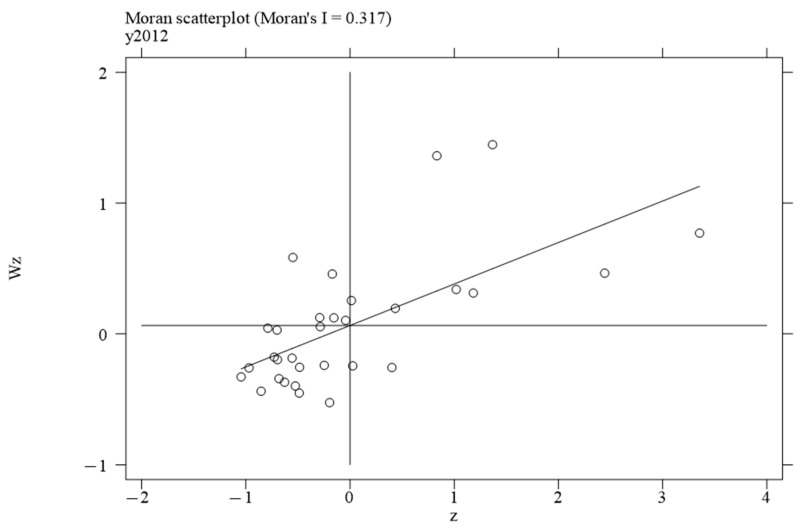
Moran scatterplot of digital infrastructure in 2012.

**Figure 4:**
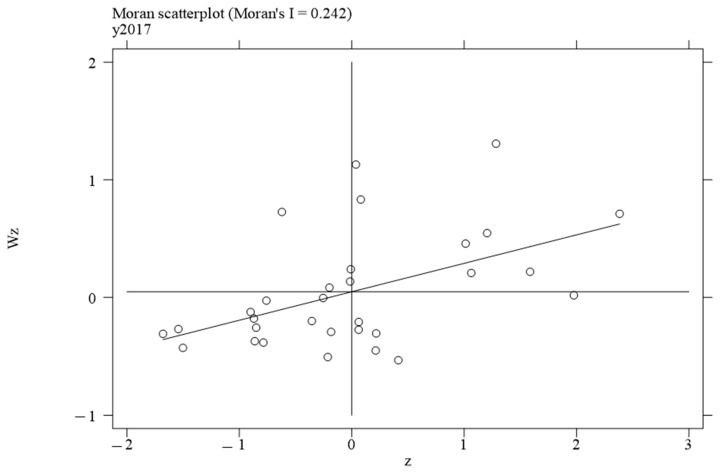
Moran scatterplot of digital infrastructure in 2017.

**Figure 5:**
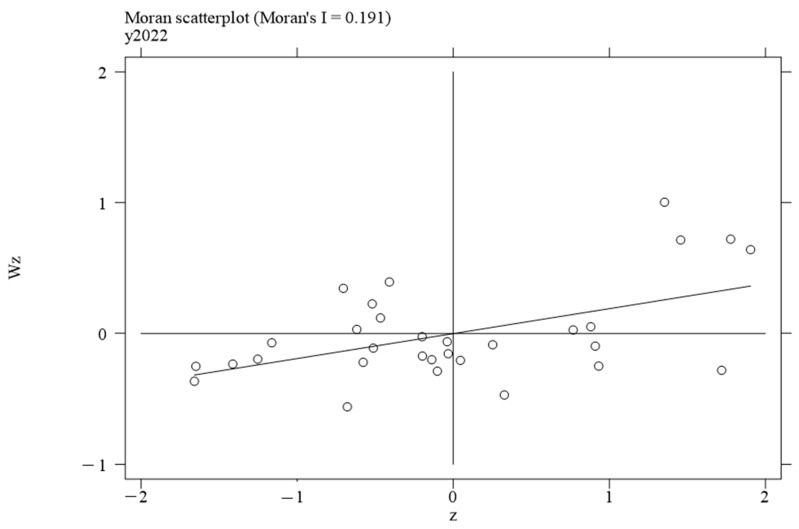
Moran scatterplot of digital infrastructure in 2022.

Figure 2, Figure 3, Figure 4 and Figure 5 report the spatial distribution of digital infrastructure in 2006, 2012, 2017, and 2022. As shown in Figure 2, Figure 3, Figure 4 and Figure 5, from 2006 to 2012, the local Moran index of digital infrastructure showed an increasing trend. The 31 provinces were mainly concentrated in the first and third quadrants, presenting a distribution pattern of “H–H” (high–high) agglomeration and “L–L” (low–low) agglomeration, indicating an enhanced spatial autocorrelation of digital infrastructure. Although the local Moran’s index of digital infrastructure in 2017 and 2022 was significantly positive, it showed a downward trend. The spatial agglomeration of the 31 provinces weakened, indicating that digital infrastructure development became more balanced in the later periods of the sample time. This may be due to the catch-up effect between economically underdeveloped regions and economically developed regions, resulting in an overall decrease in spatial agglomeration.

**Figure 6:**
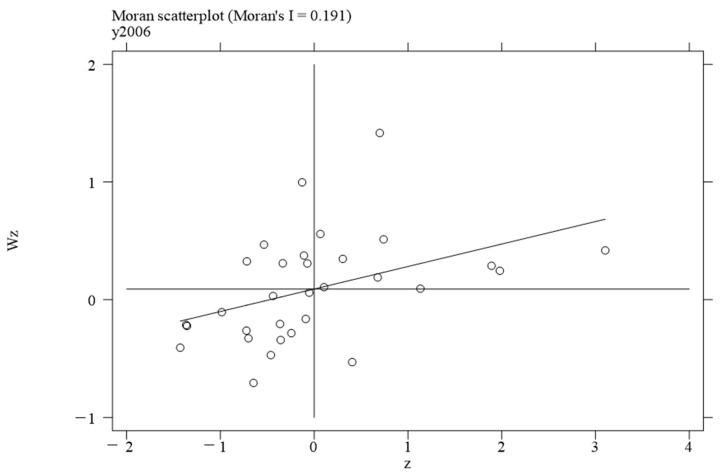
Moran scatterplot of food system resilience in 2006.

**Figure 7:**
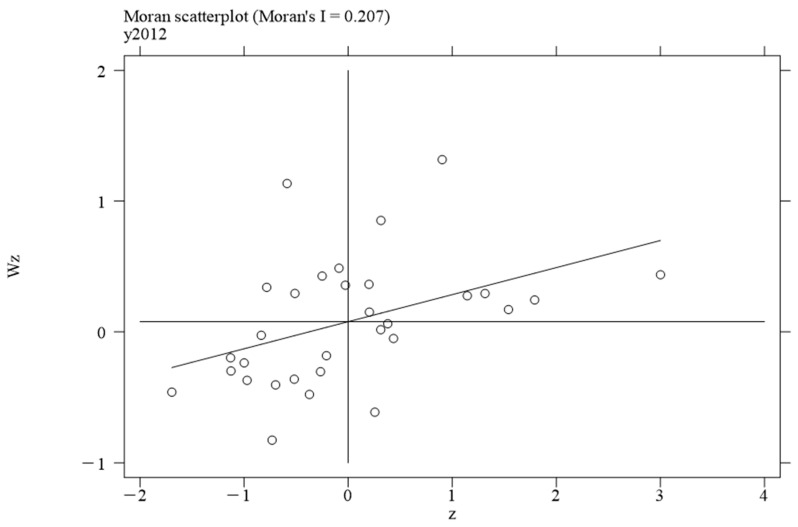
Moran scatterplot of food system resilience in 2012.

**Figure 8:**
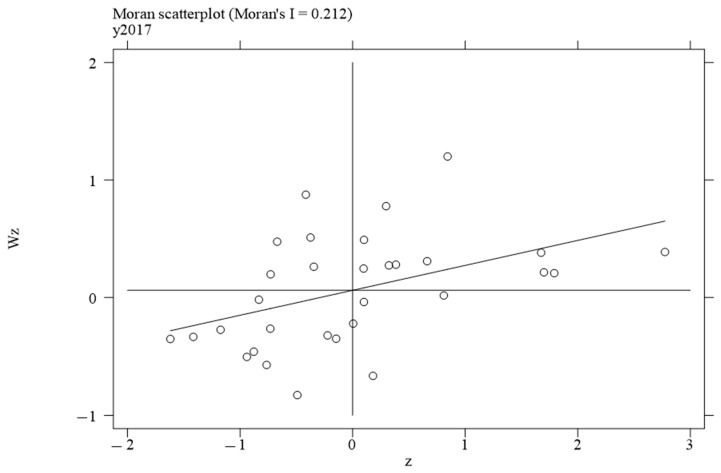
Moran scatterplot of food system resilience in 2017.

**Figure 9:**
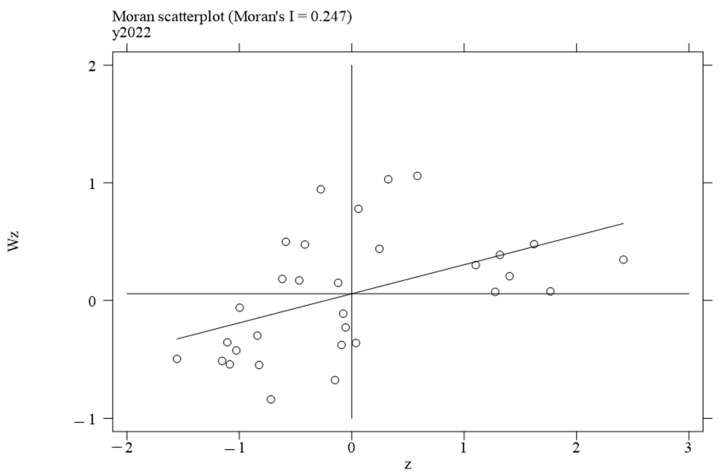
Moran scatterplot of food system resilience in 2022.

Figure 6, Figure 7, Figure 8 and Figure 9 report the spatial distribution of food system resilience in 2006, 2012, 2017, and 2022. As shown in Figure 6, Figure 7, Figure 8 and Figure 9, most of the 31 provinces were concentrated in the first and third quadrants, presenting a distribution pattern of “H–H” (high–high), “H–L” (high–low), and “L–L” (low–low) agglomeration. Moreover, the local Moran’s index of food system resilience showed an increasing trend, indicating that the spatial agglomeration of food system resilience was gradually strengthening. This finding suggests uneven regional development in food system resilience. This unevenness may be because the continuous improvement of transportation and communication infrastructure has gradually reduced the barriers to talent mobility, market access, and information among different provinces, thereby weakening the spatial agglomeration effect brought by geographic location and distance.

### 4.2. The Rationality of the Model

Table 4 reports the results of the suitability tests for the spatial model specification. As shown in Table 4, the LM test results indicate that the statistics of Moran’s I, LM-Err, LM-Lag, and Robust LM-Lag all significantly reject the null hypothesis, whereas the robust LM-Err statistic does not show statistical significance. Furthermore, the LR and Wald test statistics are both significant at the 1% significance level, rejecting the null hypothesis that the spatial Durbin model will degenerate into a spatial lag model or a spatial error model. Therefore, it is more reasonable to choose the spatial Durbin model. Meanwhile, the Hausman test result significantly rejects the null hypothesis of using the random effects at the 1% significance level. The LR test statistics of individual effects, time effects, and two-way fixed effects all significantly reject the null hypothesis at the 1% significance level, indicating that the spatio-temporal two-way fixed effects should be adopted for regression analysis. Therefore, this study ultimately used the spatial Durbin model with spatio-temporal two-way fixed effects to analyze food system resilience.

### 4.3. Regression Results

Using Stata 17.0 software, this study further analyzed the spatial impact of digital infrastructure on food system resilience. The empirical results are presented in Table 5. The results show that the value of the spatial autoregressive coefficient is 0.3579, which is significant at the 1% significance level. This finding indicates that the food system resilience of the local area is affected by that of neighboring areas. Further examination revealed that the regression coefficients of digital infrastructure (Dig) and the spatial lag term W × Dig are both significantly positive, passing the significance tests at the 5% and 1% levels, respectively. This suggests that the development of digital infrastructure not only has a positive effect on the food system resilience of the local area but also enhances the food system resilience of neighboring areas through a positive spatial spillover effect.

To further clarify the real direct and spillover effects of digital infrastructure on food system resilience, the partial differentiation decomposition method was adopted to further decompose the total effect of digital infrastructure into direct effects, indirect effects, and total effects. The results are shown in Table 5. It can be seen that for every 1 percentage point increase in digital infrastructure, digital infrastructure directly promotes the improvement of the food system resilience of the local area by 0.0443 percentage points and that of neighboring areas by 0.3060 percentage points, thereby jointly promoting the overall improvement of food system resilience by 0.3503 percentage points. This demonstrates a strong direct promoting effect and spatial spillover effect, with the spatial spillover effect accounting for 87.35% of the total effect. One possible reason for this result is that the networked characteristics of digital technology enable the synergistic effects across regions to far exceed local impacts. On the one hand, digital infrastructure (such as agricultural IoT and remote sensing monitoring) can eliminate information silos, enhance the efficiency of information exchange, and enable real-time sharing of cross-regional production data (soil moisture conditions, meteorological warnings, and pest and disease dynamics). This facilitates the establishment of a regional joint prevention and control system. It also enhances the transparency of food supply, promotes the efficiency of cross-regional food allocation, and reduces the risk of localized shortages. On the other hand, digital technologies (such as smart agricultural machinery and digital platforms) optimize resource allocation and form cross-regional collaborative networks. For example, the BeiDou Navigation Satellite System coordinates joint harvesting operations across multiple regions, reducing the idle rate of agricultural machinery. At the same time, digital platforms accelerate the diffusion of agricultural technologies. For instance, online training enables advanced planting models to rapidly radiate to surrounding areas. Based on this, Hypothesis 1 is validated.

### 4.4. Endogeneity Treatment

Numerous factors affect food system resilience, and endogeneity bias may occur due to omitted variables or bidirectional causality issues. This study employed two methods to alleviate endogeneity. One was the dynamic spatial Durbin model, as it can be used to address endogeneity problems caused by the time, space, and spatio-temporal lag terms of the dependent variable, as well as omitted variables [59]. The other was the generalized spatial two-stage least squares (GS2SLS) method. The GS2SLS model does not directly consider the time lag effect but mainly addresses endogeneity issues through instrumental variables [60]. For the GS2SLS model, following the approach of Wang J. et al. (2023) [61], the lagged dependent variable of the previous period was introduced into the model as an instrumental variable for regression. The model estimation results are presented in Table 6.

The results of the dynamic spatial Durbin model show that the spatial autoregressive coefficient is significantly positive at the 5% significance level, consistent with the previous research conclusions. In terms of time lag, the coefficient of the spatial lag term of food system resilience is 1.0164 and is significantly positive at the 1% significance level. This finding indicates that food system resilience improvement in the previous period promotes the current improvement of food system resilience, demonstrating a time cumulative effect. In terms of spatial lag, the coefficient of the spatial lag term of food system resilience is significantly negative, indicating that the food system resilience of neighboring regions in the previous period has a negative impact on the current region’s food system resilience. In other words, if the food system resilience of neighboring regions in the previous period is low, meaning they are less stable or recover more slowly in the face of external shocks, then the food system resilience of the current region may also be low.

The results of the GS2SLS model show that when not directly considering the time lag effect, the spatial autoregressive coefficient is 0.3919, and the coefficient of the lagged term of food system resilience by one period is 0.3752. Both of these findings are significantly positive at the 1% significance level. This indicates that China’s food system resilience is in a stage of steady improvement, demonstrating good development extensibility. In addition, the regression coefficients of digital infrastructure are all significantly positive at the 1% significance level, indicating that the conclusion that digital infrastructure promotes the improvement of food system resilience still holds.

### 4.5. Robustness Tests

To further verify the reliability of the research conclusions, the following methods were used for robustness testing: (1) Replace the explanatory variables. The number of internet users per 10,000 people in rural areas was used to measure digital infrastructure, replacing the core explanatory variable for robustness testing. (2) Shorten the time window. The “Broadband China” strategy was implemented in 2013. This program promoted the rapid development of digital infrastructure and, in particular, increased the Internet penetration rate in rural areas. To more accurately capture the impact of digital infrastructure on food system resilience, the time window was shortened to 2014–2022. (3) Treat the outliers. Given that individual outliers may affect the regression results, all data were re-regressed after being winsorized at the 1% quantile on both sides. (4) Replace the spatial weight matrix. The adjacency weight matrix and the spatial geographical distance weight matrix are common matrix constructions for studying spatial spillover effects. Therefore, the impact of digital infrastructure on food system resilience was re-estimated based on the adjacency weight matrix and the spatial geographical distance matrix. Columns 1 to 5 of Table 7 report the test results for replacing the explanatory variables, shortening the time window, outlier treatment, the adjacency weight matrix, and the spatial geographic distance matrix, respectively. As shown in Table 7, regardless of the method used, including replacing the explanatory variables, reducing the sample size, treating outliers, or replacing the spatial weight matrix, the spatial autocorrelation coefficient of food system resilience is significantly positive. Moreover, the direct effect and spatial spillover effect of digital infrastructure on food system resilience are both significantly positive at the 1% and 5% significance levels. This finding is consistent with the previous regression results and confirms the robustness of the basic conclusions.

### 4.6. Regional Heterogeneity Analysis

Significant differences in natural conditions, population distribution, technological conditions, transportation convenience, economic development, and institutional environments are noted among different regions. These differences may affect the promoting effect of digital infrastructure on food system resilience. Therefore, the Hu Line was used as the regional demarcation standard to divide the entire sample into the southeast region and the northwest region, and group regressions were conducted on the samples. The regression results are shown in Table 8. According to Table 8, the spatial autoregressive coefficient of food system resilience is significantly positive in the southeast region and significantly negative in the northwest region. This finding indicates substantial differences in food system resilience between the southeast and northwest regions.

Further analysis from the perspective of effect decomposition revealed the following: In terms of the direct effect, the regression coefficient of digital infrastructure in the southeast region is 0.0420, which is significant at the 1% level, whereas the regression coefficient in the northwest region is 0.0997, which is significantly positive at the 1% level. This finding indicates that the direct effect of digital infrastructure on food system resilience is more pronounced in the northwest region. In terms of the indirect effect and the total effect, the regression coefficients of digital infrastructure in the southeast region are significantly higher than those in the northwest region, suggesting that the influence of digital infrastructure in neighboring areas on local food system resilience is more significant in the southeast region. The possible reasons are as follows:

The southeast region has a more developed economy and more advanced digital infrastructure. For instance, the 5G network coverage rate is higher in this region. Due to the implementation of regional integration policies (such as the Yangtze River Economic Belt strategy), the connections among provinces within the region are closer, and the flows of factors such as technology, capital, and information are relatively frequent. Meanwhile, the dense population in the southeast region facilitates the cross-provincial diffusion of the network effect of digital infrastructure, further amplifying the spillover effect of technology. This, subsequently, makes the indirect effect of digital infrastructure more significant, and the spillover effect, combined with the direct effect, magnifies the overall impact.

The northwest region has a larger number of large-scale state-owned farms (e.g., the Xinjiang Production and Construction Corps, which shoulders the responsibility of ensuring national food security). These economic entities are more likely to adopt and apply digital technologies to improve food production efficiency and address risks. Digital infrastructure can better serve large-scale, organized production in this region. Additionally, the northwest region features an arid and variable climate, with scarce and unevenly distributed precipitation. Digital infrastructure plays a crucial role in the development of precision agriculture. For example, by leveraging digital technologies such as satellite remote sensing, extreme weather conditions can be monitored and predicted in advance, enabling timely deployment and reducing the impact of disasters. However, the northwest region is vast and sparsely populated, with weaker economic ties among provinces. The diffusion of digital technology is constrained by distance and progresses at a slower pace. Moreover, the policies in the northwest region are more focused on increasing farmers’ income and ecological protection, with fewer cross-regional collaborations. As a result, the direct effect of digital infrastructure on food system resilience dominates, whereas the indirect effect is relatively weaker.

## 5. Further Analysis

### 5.1. Threshold Effect Analysis

Digital infrastructure is conducive to improving food system resilience. Market integration reflects the degree of free flow of regional factors, which affects resource allocation efficiency and industrial structure optimization. It also provides a favorable environment for digital infrastructure to play a role [62]. Therefore, this study selected market integration as the threshold variable to further explore whether the impact of digital infrastructure on food system resilience is constrained by market integration. Referring to the research of Hansen (1999) [63], a panel single-threshold model was constructed, as follows:

(12) $$resi_{it}=\alpha +\delta_{1}dig_{it}\times I\left(market\leq \theta_{1}\right)+\delta_{2}dig_{it}\times I\left(market>\theta_{2}\right)+\gamma controls_{it}+\mu_{i}+\lambda_{i}+\varepsilon_{it}$$

**Figure 10:**
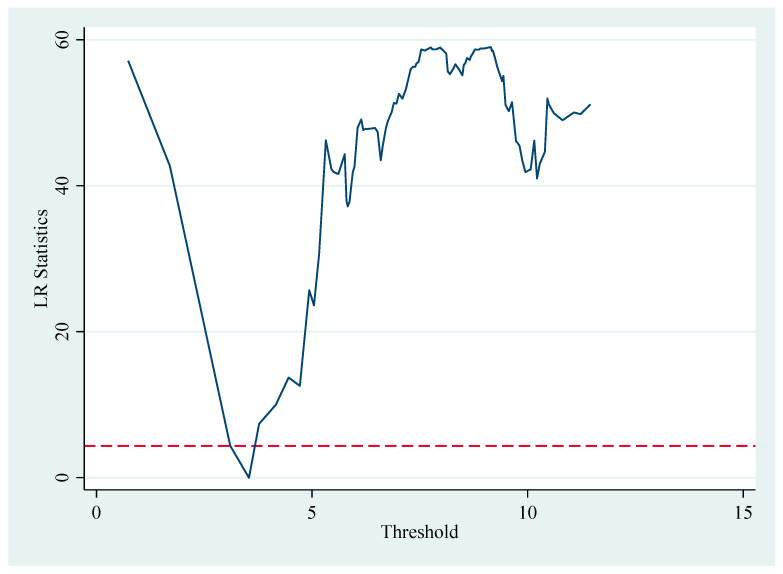
Trend of the single-threshold estimation value.

Here, Market represents the threshold variable, namely market integration, and θ represents the threshold value. Referring to the research of Zhao Y.L. et al. (2011) [64], the Fan Gang index was used as the proxy indicator of market integration to reflect the degree of marketization of regions. Specifically, the threshold regression results were obtained after 500 samplings using the Bootstrap method (Table 9). The regression results show that market integration passes the 1% significance test in the single-threshold test. However, the *p*-value for the double-threshold test is 0.2040, indicating a lack of significance. The trend chart of the threshold estimate is presented in Figure 10, which shows that the LR statistic corresponding to the threshold value (3.3100) falls below the 5% critical value and approaches 0. Therefore, market integration can be divided into two stages: market ≤ 3.3100 and market > 3.3100. This finding indicates that there is a threshold effect of market integration on the impact of digital infrastructure on food system resilience. Specifically, the panel threshold regression results are shown in Table 9.

According to Table 9, after adding control variables, when market integration is below the threshold value (market < 3.3100), the estimated coefficient of the impact of digital infrastructure on food system resilience is 0.2242. This is significant at the 1% significance level. When market integration is above the threshold value (market > 3.3100), the estimated coefficient is 0.0790. This is also significant at the 1% significance level, but its regression coefficient decreases significantly. This result indicates that the impact of digital infrastructure on food system resilience shows a marginal diminishing phenomenon as the marketization degree increases.

Potential reasons include the following: When the degree of market integration is relatively low, regional market segmentation is relatively serious, and numerous barriers, such as administrative, institutional, information, and technological barriers, potentially limit the free flow of resources and factors between regions. At this time, the development of digital infrastructure can further overcome these barriers, promote coordinated development among regions, and facilitate communication and cooperation among upstream and downstream enterprises in the grain industry chain, thus improving food system resilience. When the degree of market integration is relatively high, regional market segmentation is alleviated, and various barriers are overcome to a certain extent, enabling resources and factors to flow effectively between regions. As a result, the promoting effect of digital infrastructure on food system resilience gradually weakens. Accordingly, Hypothesis 3 is verified.

### 5.2. Analysis of the Decay Boundary of Spatial Spillover Effects

**Figure 11:**
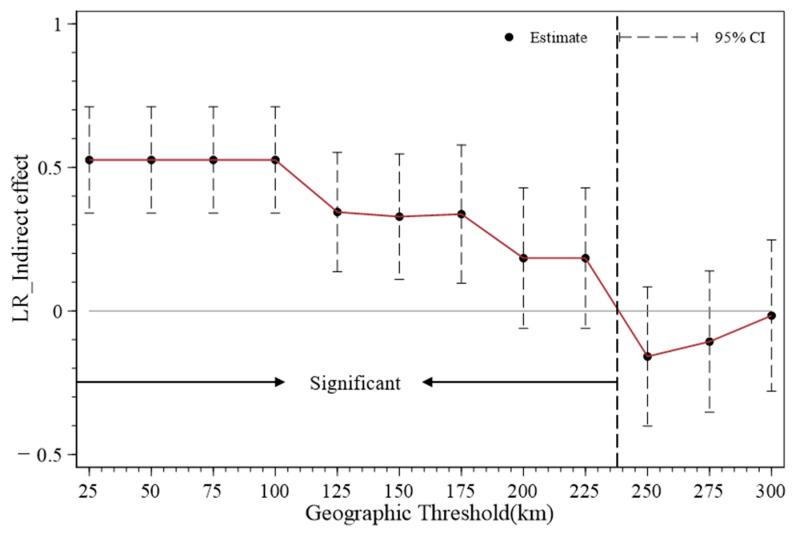
Spillover effects of digital infrastructure on food system resilience within different spatial distance ranges.

The first law hypothesis of geographical spatial relationships posits that spatial spillover effects will continuously diminish as the distance between regions increases. To further investigate the spatial attenuation boundary of the spillover effect of digital infrastructure on the resilience of the grain system, this study took 25 km as the base period and conducted regression at intervals of 25 km using the spatio-temporal double-fixed spatial Durbin model based on the spatial inverse distance matrix. Given that the spatial regression results of the spillover effect of digital infrastructure on the resilience of the grain system were not significant after 225 km, 25, 50, 75, 100, 125, 150, 175, 200, 225, 250, 275, and 300 km were ultimately selected as threshold distances. In addition, the estimated coefficients of the spillover effect of digital infrastructure on the resilience of the grain system within different spatial distance ranges are recorded and presented in Figure 11. According to Figure 11, when the distance is within 225 km, the estimated coefficients of the indirect effects of digital infrastructure are all positive and significant at least at the 10% significance level. When the distance exceeds 225 km, the estimated coefficients of the indirect effects of digital infrastructure are all negative and insignificant, indicating that the spatial effective boundary of the spillover effects of digital infrastructure on food system resilience is 225 km. Potential reasons for this finding are provided below.

Within a short distance, digital infrastructure not only plays an interconnection role efficiently, significantly breaking down information barriers and reducing information frictions, but also facilitates the application of digital technologies in the agricultural field, reducing enterprise transaction costs [65]. It can promote the free flow of production factors, knowledge, and advanced experiences such as management within regions or industries and accelerate the digital and intelligent transformation of the entire agricultural industry chain [66], thus facilitating the coordinated development of various links in the regional and grain industries. Therefore, within 225 km, the spillover effects of digital infrastructure on the food system resilience of neighboring regions mainly show the benefits of positive externalities. When the distance exceeds 225 km, expenses such as information, transportation, and transaction costs increase with the extension of geographical distance, weakening the close connection of knowledge and technology among different regions. Meanwhile, administrative and institutional barriers existing among provinces may impede the effective spatial flow of production factors and advanced experiences, thus leading to the gradual attenuation of the spatial spillover effect of digital infrastructure on the resilience of the grain system in neighboring regions.

## 6. Discussion

Existing studies have reached a consensus that digital technologies play an important role in the food system. For example, Brassesco et al. suggest that digital technologies can promote the transformation of the food system by influencing food production, logistics management, and early risk warning capabilities [67]. Khatami et al. report that digital platforms and online shopping methods make important contributions to enhancing the resilience of the food system [68]. This study showed that digital infrastructure has a significant spatial spillover effect on food system resilience, and this conclusion is similar to that of existing studies [48]. However, the difference is that this study further analyzed regional heterogeneity. Due to differences in economic development levels, transportation infrastructure, and digital infrastructure, the spatial spillover effect of digital infrastructure on food system resilience shows different impacts in different regions. In addition, this study analyzed the attenuation boundary of the spatial spillover effect of digital infrastructure on food system resilience; that is, as the geographical distance increases, the spatial spillover effect of digital infrastructure gradually weakens. This is mainly because geographical distance weakens the connection between digital infrastructure and various links such as food production, processing, or sales. Moreover, once the distance becomes farther, the increase in food transportation and information dissemination costs will lead to a weakening or even insignificance of the spillover effect of digital infrastructure. This study also found that the impact of digital infrastructure on food system resilience is constrained by the degree of market integration. When the degree of market integration increases, the economic benefits of digital infrastructure will gradually weaken. This analysis addressed the limitations of existing studies that overlooked the nonlinear impact of digital infrastructure on food system resilience [49] and further clarified the relationship between digital infrastructure and food system resilience.

### 6.1. Conclusions

Exploring how digital infrastructure affects food system resilience is not only conducive to enhancing the understanding of its role in modern agricultural development but also provides a theoretical reference for strengthening food system resilience and ensuring food security. This study used provincial panel data from 2006 to 2022 and employed the spatial Durbin model to investigate the impact of digital infrastructure on food system resilience. The following conclusions are drawn: First, digital infrastructure not only has a positive effect on local food system resilience but also promotes improvements in food system resilience in neighboring regions. It has a strong direct promoting effect and spatial spillover effect. Additionally, the impact of digital infrastructure on food system resilience exhibits significant regional heterogeneity. The direct effect of digital infrastructure on food system resilience is more significant in the northwest region, whereas the indirect and total effects are more pronounced in the southeast region. Second, the impact of digital infrastructure on food system resilience shows a threshold effect of market integration, and its role weakens as the degree of market integration increases. Third, the spillover effect of digital infrastructure on food system resilience is significant within a range of 225 km, but it gradually attenuates as geographical distance increases.

### 6.2. Implications

Based on these findings, this study proposes the following suggestions: build a food industry chain information sharing platform, integrate data resources in the food industry chain, promote the informatization management of the entire process of the food industry chain supply chain, facilitate data sharing and interconnection, achieve resource sharing and business collaboration in the food industry chain, and enhance the competitiveness and resilience of the food industry chain. Other suggestions include strengthening the construction of the digital technology talent team according to the needs of the food industry, as well as cultivating and introducing a group of scientific and professional talents in the digital field. The construction of digital facilities, such as satellite remote sensing monitoring, intelligent robots, industrial internet, and sensor terminals, should be actively promoted. Efforts should be made to continue to develop application scenarios of digital technologies in the food industry, promote the digital transformation of the entire food chain of “production, purchase, storage, processing, and sales,” reduce the adverse impact of force majeure factors on the food industry, and thus enhance the resilience of the food industry chain.

Considering that the impact of digital infrastructure on the resilience of the food industry chain exhibits regional heterogeneity and that its spatial spillover effect attenuates as geographical distance increases, it is necessary to optimize the construction of digital infrastructure according to local conditions. In the southeast region, it is necessary to further strengthen regional digital governance cooperation, form a regional digital technology application cluster, and release the spatial spillover effect of digital infrastructure. In the northwest region, priority should be given to improving digital infrastructure. Potential measures include introducing digital technology through models such as “enclave economy”; strengthening exchanges, cooperation, and information sharing in aspects such as agricultural technology promotion and digital talent cultivation; minimizing the regional imbalance of digital infrastructure; bridging the digital divide; and expanding the spatial spillover boundary of the effect of digital infrastructure on food system resilience.

Moreover, it is important to guard against the risks of excessive concentration in market integration. On the one hand, it is critical to clarify the ownership of data such as grain planting data and transaction records, establish a regional data trading platform, and allow small and medium-sized participants to obtain benefits through data sharing. On the other hand, for emerging fields such as grain trading platforms and smart agriculture services, it is important to formulate anti-monopoly guidelines to prohibit the abuse of market dominance.

### 6.3. Research Limitations

This study provides empirical evidence that digital infrastructure can enhance food system resilience. However, there are certain limitations. First, there are limitations in terms of data. The current study mainly used provincial panel data. Although provincial panel data can reflect macro trends, they fail to capture some behaviors and decisions of consumers and producers. For example, factors such as education level, income level, and consumption habits can affect food supply and demand. Second, there are limitations in the scope of the study. The current research focused more on the production and supply aspects of the food system, while giving relatively insufficient consideration to factors on the demand side. However, due to the difficulty in obtaining data, we did not incorporate food demand factors into the research framework. Thus, this study was unable to fully reveal the true performance of food system resilience in the context of supply and demand interactions.

In response to the above-mentioned research limitations, future research could attempt to integrate micro-level data, such as education level, income level, and consumption preferences. By combining macro- and micro-level data, a more comprehensive understanding of the resilience of the food system can be achieved. Additionally, future research could strengthen the study of food demand. Data related to food demand, such as the amount of food consumption, consumption structure, and consumption frequency of individuals or households, could be collected through methods such as questionnaires and field interviews. The impact of demand changes on the resilience of the food system could then be analyzed.

## Figures and Tables

**Table 1:** Indicator system of food system resilience.

Primary Indicators	Secondary Indicators	Indicator Calculation Method	Direction	Weight	Data Sources
Resistance ability (0.6449)	Basic guarantee (0.1666)	Total cultivated land area/total population at the end of the year	+	0.0412	China Statistical Yearbook
		Grain output value/rural population	+	0.0770	National Bureau of Statistics of China
		Effective irrigated area × a	+	0.0484	National Bureau of Statistics of China
	Supply stability (0.2773)	Total grain output/total grain sown area	+	0.0117	National Bureau of Statistics of China
		Total grain output/total population	+	0.0363	National Bureau of Statistics of China
		Density of grade highways	+	0.1258	National Bureau of Statistics of China
		Employees in road, railway, and air transportation industries × (employees in primary industry/total employees in the whole society)	+	0.0260	China Statistical Yearbook of the Tertiary Industry
		Purchase–sale difference of raw grains for state-owned grain enterprises	−	0.0010	National Bureau of Statistics of China
		Stability of grain commodity consumption prices	+	0.0765	National Bureau of Statistics of China
	Risk control (0.2010)	Number of grain emergency supply outlets	+	0.0433	National Bureau of Statistics of China
		Premium income/total output value of agriculture, forestry, animal husbandry, and fishery	+	0.0729 0.0848	EPS DATA
		Premium income/total rural population	+		EPS DATA
Recovery ability (0.0420)	Recoverability (0.0374)	Total sown area of crops throughout the year/total cultivated land area	+	0.0177	National Bureau of Statistics of China
		(Current total agricultural output value—previous total agricultural output value)/previous total agricultural output value	+	0.0035	National Bureau of Statistics of China
		Per capita disposable income of rural residents	+	0.0030	National Bureau of Statistics of China
		Average number of health clinic staff per village	+	0.0132	National Bureau of Statistics of China
	Sustainability (0.0045)	Quantity of chemical fertilizers applied in agriculture/total sown area of crops × a	−	0.0003 0.0003	China Rural Statistical Yearbook
		Quantity of pesticides applied/total sown area of crops × a	−		China Rural Statistical Yearbook
		Area of crops affected by disasters/total sown area of crops × a	−	0.0040	National Bureau of Statistics of China
Transformation ability (0.3132)	Industrial synergy (0.1942)	Total output value of agricultural, forestry, animal husbandry, and fishery services/total output value of agriculture, forestry, animal husbandry, and fishery	+	0.0144	China Statistical Yearbook of the Tertiary Industry
		Number of agricultural product processing enterprises/rural population	+	0.0472	China Academy for Rural Development-Qiyan China Agri-research Database (CCAD), Zhejiang University
		Total output value of grain and oil processing industry/total agricultural output value	+	0.0519	EPS DATA
		Number of professional cooperatives between farmers/rural population	+	0.0808	China Rural Cooperative Management Statistical Annual Report China Rural Cooperative Economy Statistical Annual Report
	Innovation synergy (0.0710)	Funds for agricultural science and technology activities/employees in primary industry	+	0.0710	China Population and Employment Statistical Yearbook
	Government synergy (0.0107)	Expenditure on agriculture, forestry, and water affairs/fiscal expenditure × b	+	0.0107	China Fiscal Yearbook
	Financial synergy (0.0373)	Agricultural loans × b	+	0.0373	EPS DATA

a = Grain sown area/total sown area of crops; b = agricultural output value/output value of agriculture, forestry, animal husbandry, and fishery. In addition, to make the table more aesthetically pleasing, the data in the table are uniformly expressed to 4 decimal places.

**Table 2:** Descriptive statistics of the variables.

Variable Name	Variable Symbols	Obs	Mean	SD	Min	Max
Food system resilience	Resi	527	0.1600	0.0680	0.0410	0.4170
Digital infrastructure	Dig	527	0.3770	0.2760	0.0170	1.0750
Rural population aging	Aging	527	0.1190	0.0440	0.0500	0.2750
Level of agricultural economic development	Adev	527	10.4170	5.5310	0.2000	30.2000
Grain fixed asset investment	Inve	527	9.4680	12.0980	0.0000	82.6270
Rural electricity consumption	Elec	527	0.1770	0.5210	0.0030	4.8670
Educational attainment of rural residents	Educ	527	7.6000	0.8670	3.8190	10.1150

**Table 3:** Global Moran’s index of digital infrastructure and food system resilience.

Year	Digital Infrastructure		Food System Resilience	
	I	*p*-Value	I	*p*-Value
2006	0.2630	0.0000	0.1910	0.0080
2007	0.2710	0.0000	0.1270	0.0600
2008	0.2670	0.0000	0.1330	0.0490
2009	0.2700	0.0000	0.1400	0.0440
2010	0.2910	0.0000	0.1520	0.0270
2011	0.3740	0.0000	0.1830	0.0120
2012	0.3170	0.0000	0.2070	0.0050
2013	0.2140	0.0030	0.1630	0.0230
2014	0.2090	0.0050	0.1890	0.0100
2015	0.1650	0.0210	0.1690	0.0190
2016	0.2240	0.0030	0.1920	0.0090
2017	0.2420	0.0020	0.2120	0.0050
2018	0.1530	0.0330	0.2080	0.0060
2019	0.2190	0.0040	0.2190	0.0040
2020	0.2320	0.0030	0.2270	0.0030
2021	0.1470	0.0410	0.2570	0.0010
2022	0.1910	0.0110	0.2470	0.0010

**Table 4:** Test results for the selection of spatial models.

	Test	Statistic	*p*-Value
Spatial error	Moran’s I	4.2080	0.0000
	LM-err	5.7600	0.0016
	Robust LM-err	0.8400	0.3590
Spatial lag	LM-lag	66.3030	0.0000
	Robust LM-lag	61.3830	0.0000
Hausman Test		68.11	0.0000
Wald test	Degeneration into SAR	58.64	0.0000
	Degeneration into SEM	65.27	0.0000
LR test	Degeneration into IND	41.61	0.0000
	Degeneration into TIME	1032.39	0.0000
LR test	Degeneration into SAR	52.99	0.0000
	Degeneration into SEM	54.18	0.0000

**Table 5:** Regression results of the spatial Durbin model.

Variable	Main	W	Direct Effect	Indirect Effect	Total Effect
Dig	0.0310 **	0.1942 ***	0.0443 ***	0.3060 ***	0.3503 ***
	(0.0124)	(0.0337)	(0.0135)	(0.0592)	(0.0659)
Aging	−0.1561 ***	0.6428 ***	−0.1218 **	0.8711 ***	0.7493 ***
	(0.0512)	(0.1385)	(0.0475)	(0.1922)	(0.1914)
Adev	0.0015 ***	−0.0023 *	0.0015 ***	−0.0025	−0.0010
	(0.0005)	(0.0012)	(0.0005)	(0.0018)	(0.0020)
Inve	0.0005 ***	0.0006 *	0.0006 ***	0.0013 **	0.0019 ***
	(0.0002)	(0.0004)	(0.0002)	(0.0005)	(0.0006)
Elec	−0.0058 ***	−0.0032	−0.0061 ***	−0.0082	−0.0143 *
	(0.0021)	(0.0051)	(0.0021)	(0.0076)	(0.0084)
Educ	−0.0034	0.0169	−0.0023	0.0249	0.0227
	(0.0039)	(0.0111)	(0.0039)	(0.0175)	(0.0189)
Year	Yes				
Province	Yes				
Spatial rho	0.3579 ***				
	(0.0692)				
*N*	527				
*R* ^2^	0.4319				

* *p* < 0.1, ** *p* < 0.05, *** *p* < 0.01.

**Table 6:** Test results based on the DSDM and GS2SLS models.

Variable	DSDM		GS2SLS
	Main	W	
L. Resi	1.0164 ***		
	(0.0117)		
L.W. Resi	−0.1510 *		0.3752 ***
	(0.0853)		(0.1243)
Dig	0.0205 ***	0.0818 ***	0.0527 ***
	(0.0056)	(0.0072)	(0.0159)
Aging	−0.0449 ***	0.0859 *	0.4629 ***
	(0.0171)	(0.0455)	(0.0561)
Adev	0.0001	0.0013 **	0.0004
	(0.0001)	(0.0005)	(0.0003)
Inve	0.0001	0.0011 ***	−0.0002 *
	(0.0001)	(0.0002)	(0.0001)
Elec	−0.0054 ***	−0.0006	−0.0113 ***
	(0.0011)	(0.0024)	(0.0027)
Educ	0.0001	0.0187 ***	−0.0030
	(0.0007)	(0.0039)	(0.0023)
Spatial rho	0.1696 **		0.3919 ***
	(0.0770)		(11.484)
*N*	496		527
*R* ^2^	0.8772		0.5341

* *p* < 0.1, ** *p* < 0.05, *** *p* < 0.01.

**Table 7:** Robustness test.

Variable	(1)	(2)	(3)	(4)	(5)
Dig	0.0103 **	0.0335 ***	0.0314 **	0.0392 ***	0.0457 ***
	(0.0040)	(0.0125)	(0.0125)	(0.0127)	(0.0129)
Aging	−0.2070 ***	−0.0768	−0.2158 ***	−0.1005 *	−0.1467 ***
	(0.0507)	(0.0624)	(0.0512)	(0.0518)	(0.0498)
Adev	0.0024 ***	−0.0020 **	0.0014 ***	0.0013 ***	0.0013 **
	(0.0005)	(0.0010)	(0.0005)	(0.0005)	(0.0005)
Inve	0.0005 ***	0.0002	0.0006 ***	0.0005 ***	0.0006 ***
	(0.0002)	(0.0002)	(0.0002)	(0.0002)	(0.0002)
Elec	−0.0045 **	−0.0052 ***	−0.0053 **	−0.0016	−0.0056 ***
	(0.0021)	(0.0019)	(0.0025)	(0.0021)	(0.0021)
Educ	−0.0079 **	0.0146 ***	−0.0102 **	0.0026	−0.0018
	(0.0039)	(0.0044)	(0.0041)	(0.0041)	(0.0039)
W Dig	0.0445 ***	0.1228 ***	0.1901 ***	0.3533 ***	0.4915 ***
	(0.0084)	(0.0292)	(0.0339)	(0.0721)	(0.0901)
W Aging	0.4193 ***	0.6854 ***	0.6782 ***	0.4812 **	2.2886 ***
	(0.1378)	(0.1507)	(0.1357)	(0.1974)	(0.3231)
W Adev	0.0008	0.0039 *	−0.0024 **	−0.0025	−0.0036
	(0.0011)	(0.0022)	(0.0012)	(0.0020)	(0.0031)
W Inve	0.0005	0.0000	0.0007 *	−0.0006	0.0016
	(0.0004)	(0.0004)	(0.0004)	(0.0008)	(0.0010)
W Elec	−0.0065	−0.0031	−0.0062	−0.0134	−0.0000
	(0.0050)	(0.0049)	(0.0059)	(0.0083)	(0.0127)
W Educ	−0.0029	−0.0077	0.0103	0.0012	0.0666 **
	(0.0114)	(0.0121)	(0.0113)	(0.0199)	(0.0290)
Direct effect Dig	0.0117 ***	0.0430 ***	0.0445 ***	0.0505 ***	0.0535 ***
	(0.0040)	(0.0137)	(0.0136)	(0.0145)	(0.0145)
Indirect effect Dig	0.0554 ***	0.2086 ***	0.3007 ***	0.5359 ***	0.6535 ***
	(0.0095)	(0.0594)	(0.0600)	(0.1222)	(0.1708)
Total effect Dig	0.0672 ***	0.2516 ***	0.3452 ***	0.5863 ***	0.7070 ***
	(0.0092)	(0.0666)	(0.0668)	(0.1312)	(0.1788)
Year	Yes	Yes	Yes	Yes	Yes
Province	Yes	Yes	Yes	Yes	Yes
Spatial rho	0.1871 **	0.3701 ***	0.3580 ***	0.3258 ***	0.2286 *
	(0.0798)	(0.0914)	(0.0692)	(0.0903)	(0.1369)
*N*	527	279	527	527	527
*R* ^2^	0.2627	0.2146	0.4135	0.4034	0.3880

* *p* < 0.1, ** *p* < 0.05, *** *p* < 0.01.

**Table 8:** Results of regional heterogeneity analysis.

Variable	Southeast Region		Northwest Region	
	Main	W	Main	W
Dig	0.0209 *	0.1822 ***	0.1234 ***	0.2361 **
	(0.0114)	(0.0290)	(0.0412)	(0.1032)
Aging	−0.0745	0.4772 ***	−0.0601	−0.1736
	(0.0551)	(0.1340)	(0.1191)	(0.2993)
Adev	0.0028 ***	−0.0022 **	−0.0062 ***	−0.0089 **
	(0.0005)	(0.0010)	(0.0015)	(0.0038)
Inve	0.0000	0.0009 ***	0.0010	−0.0042 *
	(0.0002)	(0.0003)	(0.0007)	(0.0025)
Elec	−0.0059 ***	−0.0016	0.2223 *	1.5960 ***
	(0.0018)	(0.0043)	(0.1255)	(0.4505)
Educ	−0.0045	0.0215 **	0.0183 ***	−0.0086
	(0.0041)	(0.0106)	(0.0060)	(0.0157)
Direct effect Dig	0.0420 ***		0.0997 ***	
	(0.0132)		(0.0376)	
Indirect effect Dig	0.3433 ***		0.1560 *	
	(0.0652)		(0.0865)	
Total effect Dig	0.3853 ***		0.2557 **	
	(0.0730)		(0.1062)	
Year	Yes		Yes	
Province	Yes		Yes	
Spatial rho	0.4733 ***		−0.4576 ***	
	(0.0627)		(0.1405)	
*N*	425		102	
*R* ^2^	0.3583		0.7177	

* *p* < 0.1, ** *p* < 0.05, *** *p* < 0.01. The northwest region includes six provinces, including Inner Mongolia, Gansu, Tibet, Qinghai, Ningxia, and Xinjiang, while the southeast region comprises the remaining 25 provinces.

**Table 9:** Estimation results of the threshold model.

	Threshold Effect Test		Single Threshold	
Single threshold	F-value	71.4400		
	*p*-value	0.0040		
Double threshold	F-value	32.5600		
	*p*-value	0.2040		
Market < 3.3100			0.2346 ***	0.2242 ***
			(0.0176)	(0.0177)
Market > 3.3100			0.1433 ***	0.0790 ***
			(0.0034)	(0.0073)
Controls			No	Yes
Con_s			0.1053 ***	−0.1694 ***
			(0.0016)	(0.0292)
*N*			527	527
*R* ^2^			0.7899	0.8445

*** *p* < 0.01.

## Data Availability

The original contributions presented in this study are included in the article. Further inquiries can be directed to the corresponding author.
